# Genome-Wide Expression Analysis Identifies a Modulator of Ionizing Radiation-Induced p53-Independent Apoptosis in *Drosophila melanogaster*

**DOI:** 10.1371/journal.pone.0036539

**Published:** 2012-05-29

**Authors:** Petra van Bergeijk, Joseph Heimiller, Lyle Uyetake, Tin Tin Su

**Affiliations:** Department of Molecular, Cellular and Developmental Biology, University of Colorado, Boulder, Colorado, United States of America; University of Chicago, United States of America

## Abstract

Tumor suppressor p53 plays a key role in DNA damage responses in metazoa, yet more than half of human tumors show p53 deficiencies. Therefore, understanding how therapeutic genotoxins such as ionizing radiation (IR) can elicit DNA damage responses in a p53-independent manner is of clinical importance. Drosophila has been a good model to study the effects of IR because DNA damage responses as well as underlying genes are conserved in this model, and because streamlined gene families make loss-of-function analyses feasible. Indeed, Drosophila is the only genetically tractable model for IR-induced, p53-independent apoptosis and for tissue regeneration and homeostasis after radiation damage. While these phenomenon occur only in the larvae, all genome-wide gene expression analyses after irradiation to date have been in embryos. We report here the first analysis of IR-induced, genome-wide gene expression changes in wild type and p53 mutant Drosophila larvae. Key data from microarrays were confirmed by quantitative RT-PCR. The results solidify the central role of p53 in IR-induced transcriptome changes, but also show that nearly all changes are made of both p53-dependent and p53-independent components. p53 is found to be necessary not just for the induction of but also for the repression of transcript levels for many genes in response to IR. Furthermore, Functional analysis of one of the top-changing genes, EF1a-100E, implicates it in repression of IR-induced p53-independent apoptosis. These and other results support the emerging notion that there is not a single dominant mechanism but that both positive and negative inputs collaborate to induce p53-independent apoptosis in response to IR in Drosophila larvae.

## Introduction

Ionizing Radiation (IR) causes double strand breaks (DSB) in the DNA, which results in three well-studied cellular responses: cell cycle regulation by checkpoints, DNA repair and apoptosis. Tumor suppressor p53 plays a key role in the induction of all three responses [1], [2]. In response to DNA DSBs, checkpoint kinases, ATM, ATR, Chk1 and Chk2, become activated. This results in phosphorylation and stabilization of p53. Transcriptional targets of p53 include cell cycle inhibitors, DNA repair genes and pro-apoptotic genes. Induction of apoptosis forms the basis for the use of IR in the treatment of cancers. Yet, a majority of human solid tumors are deficient in p53 function [3]. Therefore, understanding how IR induces apoptosis in the absence of p53 is of clinical importance. In this regard, it would be useful to understand mechanisms that activate p53-independent apoptosis as well as those that inhibit p53-independent apoptosis. Antagonists of the latter could enhance cell killing by radiation therapy, especially for p53-deficient tumors.

Mammalian p53 family comprises p53, p63 and p73 [4]. Mammalian cells that are compromised for p53 function still undergo apoptosis when exposed to IR, UV or chemotherapy agents such as topoisomerase inhibitors. p53-independent apoptosis in response to topoisomerase inhibitors is mediated by p73 that can activate the expression of pro-apoptotic genes [5]. Whether p73 is required for radiation-induced p53-independent apoptosis is not known, but available data suggest this is the case. p73 expression correlates with the level of radiation-induced apoptosis in the absence of p53 in tumor samples of human cervical cancer patients [6]. Forced expression of p73 in human vestibular schwannoma cells also sensitized cells to ionizing radiation-induced apoptosis [7].

We have reported previously that *Drosophila melanogaster* undergoes IR-induced, p53-independent, caspase-dependent apoptosis, albeit with a delay compared to wild type [8]. This makes *Drosophila* the first genetically tractable experimental model to show this mode of cell death. Moreover, there is only a single p53 homolog in Drosophila; thus, apoptosis in p53 mutant Drosophila occurs independently of all p53 family members. Therefore, any mechanism identified for p53-independent apoptosis is likely to be novel. Since the identification of IR-induced, p53-independent apoptosis in Drosophila, we and others have identified genes that modulate the level of this mode of cell death. These are *hid* (a Smac/DIABLO ortholog), *grapes* (Chk1), JNK pathway components, and E2F family of transcription factors [8], [9], [10]. Interestingly, however, while these genes either positively or negatively alter the timing and the level of p53-independent apoptosis, none is absolutely required. For example, E2F1 promotes and E2F2 represses the levels of p53-independent apoptosis, but in the absence of all E2f activities, robust p53-independent apoptosis still occurred after irradiation [10]. These data suggest that IR-induced p53-independent apoptosis is accomplished via unknown factors.

p53 homologs have non-apoptotic roles after irradiation such as induction of DNA repair. Vertebrate p53 homologs also act to arrest the cell cycle, but this function has not been seen for Drosophila p53 [11], [12], [13]. Instead, Drosophila p53 is needed for compensatory proliferation that occurs in response to apoptosis and functions to replace cells lost to cell death during larval growth [14].

All previous analyses of p53-dependent and p53-independent transcriptome have been conducted in embryos even though neither p53-independent apoptosis nor compensatory proliferation in response to radiation damage has been documented at this stage in Drosophila life cycle. Conversely, despite the known importance of p53 in radiation responses in the larvae, we do not have a comprehensive picture of the p53-dependent transcription program at this stage in Drosophila life cycle. To identify genes whose transcript levels change in response to radiation in wild type and in p53 mutants, we performed a microarray-based genome-wide expression analysis in the larvae. Because radiation responses can be tissue-specific (e.g. [15], [16]), the analysis was focused on wing imaginal discs. Genome-wide expression was compared at two different time points after irradiation, in order to address both p53-dependent and p53-independent apoptosis. The transcript level of ten candidates that showed significant changes were validated by quantitative RT-PCR. Our results corroborate the contribution of Drosophila Smac/DIABLO orthologs and the TNF/JNK pathway to p53-independent apoptosis and, in addition, identified a novel role for a translation elongation factor in this mode of cell death.

## Results

Previous studies have mapped the time course of radiation responses in larval wing imaginal discs [8], [14], [17], [18], [19]. Briefly, cell cycle arrest is in place as early as 30 min after exposure 4000 R of X-rays and persists for about 6 more hours. DNA repair is complete by about 3 hr after irradiation. Robust apoptosis is detectable at 4 hr after irradiation and continues for at least 20 more hours. In p53 mutants, apoptosis is delayed and become detectable about 18 hr after irradiation [8]. Likewise, pro-apoptotic genes such as *hid* and *rpr* that are up-regulated at 2 hr after irradiation in wild type are up-regulated at 18 hr after irradiation in p53 mutants [8]. Because of our interest in apoptosis, we chose to analyze gene expression at 2 and 18 hr after irradiation of larvae with 4000R of X-rays. mRNA was isolated from wing imaginal discs of 3^rd^ instar larvae in two independent experiments. *y^1^w^1118^* (to be called ‘*yw*’ hereafter) were used as control for *y^1^w^1118^*; *p53^5A-1-4^* homozygotes (to be called ‘*p53*’ hereafter). mRNA was hybridized to Affymetrix GeneChip Drosophila Genome 2.0 Arrays. The data quality was assessed to address the following: errors in micro-array manufacture or processing; overall probe intensity; and consistency between duplicate arrays. In brief, we found no manufacturing or processing errors, find that probe intensities and RNA degradation are similar among arrays, and found that duplicate samples give similar results (Figures S1, S2, and S3). The fact that expression changes for all 10 genes chosen for independent validation were confirmation by Q-RT-PCR (described below) attests to good data quality.

### Genome-wide changes in wild type larval wing imaginal discs

The resulting microarray data has been made accessible in two formats. First, the raw data has been deposited into the Gene Expression Omnibus (GEO) repository of the National Institutes of Health (Accession #GSE37404). Second, all genes that show significant and reproducible induction or repression (±1.5-fold or greater, p<0.005) between ANY ±IR sample pairs at 2 or 18 hr, in *yw* or *p53* discs, are supplied, along with gene ontology information, in a searchable format in Table S1. The data in Table S1, which is extensive, has been further organized into Venn diagrams (Figure S4), which are discussed in following sections.

To identify genes for further analysis, we applied a more stringent cut-off (≥2-fold, p<0.001). We first compared genes that are differentially expressed in *yw* and *p53* mutant discs without irradiation. 109 genes that are repressed and 131 genes that are activated in *p53* mutants relative to *yw* fall into Gene Ontology clusters with GO terms such as ‘hemocyanin’ and ‘storage protein’, ‘peptidase inhibitor’, ‘oxidation reduction’, ‘glutathione metabolism’ and neurogenesis' (data not shown). None are related to DNA damage responses that we are interested in. This finding and the fact that p53 null mutants are viable and fertile in the absence of genotoxins led us to focus instead on gene expression changes that occur after irradiation.

Using the same criteria (≥2-fold, p<0.001), 359 and 376 genes were induced in *yw* discs at 2 and 18 hr after IR respectively (Table S2). The two sets overlap by 230 genes (excluding 3 that are annotated as different genes but share a CG number with other genes), suggesting that induction of most genes by IR persists for several hours. These numbers translate to a 2.8% hit rate (∼360/12,948 genes on the array). Functional Annotation Clustering based on Gene Ontology identified 17 clusters that are induced at 2 hr in *yw* discs (Table 1; Enrichment Score >1.3, which corresponds to p<0.05). These included clusters of genes that function in DNA damage response, apoptosis, JNK cascade, trans-membrane transport, glutathione metabolism, proteases and regulators of proteases. The last two clusters include 25 known or predicted peptidases, only one of which is a caspase (Nedd2-like caspase). Similar analysis identified 10 clusters at 18 hr after irradiation (Table 1). The DNA damage response cluster was ranked first in both 2 hr and 18 hr samples. Cell death cluster is also found at both time points, consistent with published reports that cell death continues for at least 30 hr under these experimental conditions [8]. DNA repair cluster appeared at both time points despite published reports that DNA repair is completed by about 3 hr after irradiation [18]. We will see later that DNA repair genes, although still induced at 18 hr, are induced to a lesser degree; this can reconcile the current findings with the published work on the schedule of repair.

**Table 1:** Functional Annotation Clustering of genes induced 2-fold or greater by IR in wild type (*yw*) wing imaginal discs (p<0.001).

at 2 hr after irradiation			at 18 hr after irradiation		
rank	Enrichment Score	representative terms (GO, INTERPRO, SMART, KEGG_PATHWAY)	rank	Enrichment Score	representative terms (GO, INTERPRO, SMART, KEGG_PATHWAY)
1	5.95	**celular response to stress, response to DNA damage stimulus, DNA repair**	1	3.09	**celular response to stress, response to DNA damage stimulus, DNA repair**
2	2.97	NHEJ, DSB repair, telomere maintenance	2	2.77	CHk, CHK kinase-like
3	2.78	**positive regulation of cell death, programmed cell death, apoptosis, autophagic cell death**	3	2.17	actin cytoskeleton, actomyosin structure organization, cytoskeletal protein binding
4	2.17	**co-factor biosynthetic process, co-enzyme biosynthetic process**, oxidoreduction coenzyme metabolic process	4	2.17	**glutathione transferase activity, drug metabolism, glutathione metabolism**
5	2.17	adenyl nucleotide binding, purine nucleotide binding, ATPase activity	5	1.97	contactile fibre part, myosin II complex, actin cytoskeleton
6	1.95	**ABC transporter-like, multidrug transporter activity**	6	1.90	**co-factor biosynthetic process, co-enzyme biosynthetic process**
7	1.89	**extra-cellular matrix**	7	1.88	cell-adhesion
8	1.81	DEAD-like helicase, DNA/RNA helicase	8	1.79	**ABC transporter-like, multidrug transporter activity**
9	1.80	**glutathione transferase activity, drug metabolism, glutathione metabolism**	9	1.49	**positive regulation of cell death, programmed cell death, apoptosis, autophagic cell death**
10	1.71	salivary gland development, hemopoiesis, immune system development	10	1.39	**extra-cellular matrix**; metallopeptidase activity
11	1.68	larval development, apical part of cell			
12	1.55	apical cortex, asymmetric protein localization, cell fate commitment			
13	1.49	nucleotidyltransferase, DNA polymerase activity			
14	1.45	proteolysis, protease, peptidase activity, endopeptidase activity, hydrolase			
15	1.43	positive regulation of caspase activity, positive regulation of peptidase activity, regulation of endopeptidase activity			
16	1.33	JNK cascade, stress activated kinase signaling pathway, MAPKKK cascade, embryonic morphogenesis			

Only clusters with Enrichment Score of >1.3 are shown. Gene ontology information is from DAVID Bioinformatics Resources 6.7, NIAID/NIH (ttp://david.abcc.ncifcrf.gov/). Clusters present in both 2 hr and 18 hr time-points are in bold font.

### The effect of IR on cell death-related genes

Because we are interested in DNA damage responses and cell death, we analyzed the expression of genes in these categories as defined by GO terms in Flybase (http://flybase.org/) and in DAVID (Database for Annotation, Visualization and Integrated Discovery) Bioinformatics Resources 6.7, NIAID/NIH (ttp://david.abcc.ncifcrf.gov/) [20]. To get a more comprehensive view, we decreased the stringency (±1.5-fold or greater change, p<0.005). Table 2 shows 22 cell death-related genes that are induced by IR in *yw* at 2 hr, 18 hr or both. The list includes genes that are, according to previous studies, (i) induced by IR and (ii) needed for IR-induced apoptosis, such as *hid*, *rpr*, and *skl* (in bold font in Table 2). Also on this list are genes that promote autophagic cell death, suggesting that this form of cell death plays a role in response to IR. Most genes induced at 2 hr remained induced at 18 hr although to a lesser extent. 37 cell death-related genes that are repressed by IR in *yw* at 2 hr, 18 hr or both are shown in Table S3. Several of these are anti-apoptotic (e.g. Iap2 and Drep-1 that normally inhibits developmental apoptosis) although some are also pro-apoptotic (e.g. *mnk/lok* encoding Drosophila Chk2). These findings suggest that the expression of IR exposure affect both pro-apoptotic and anti-apoptotic genes, and that their gene products may counter-balance one another.

**Table 2:** Cell death related genes induced by IR in *yw* (≥1.5-fold, p<0.005).

	other information (Flybase)	gene	fold change (p-value)				
			y2− vs y2+	y18− vs y18+	p2− vs p2+	p18− vs p18+	p2+ vs p18+
1	DNA binding, leg morphogenesis	ftz-f1(Ftz interacting protein 1)	0.5(0.000103)	1.0(7.5e-007)	0.7(0.073569)	0.5(0.000359)	0.7(0.000122)
2	predicted DNA binding and mRNA splicing	CG6905(–)	0.6(0.000086)	0.5(0.008317)	–	–	–
3	EGF receptor binding	vn(defective dorsal discs)	0.7(0.000006)	0.6(0.000428)	-0.3(0.021632)	–	0.4(0.001491)
4	influence processing of Dredd RNA	qkr58E-3(KH domain encompassing protein 1)	0.7(4.2e-008)	0.3(0.010044)	0.6(0.000145)	–	-0.3(0.010807)
5	germ cell death	wun(wunen)	0.8(0.000671)	1.8(1.6e-008)	–	–	–
6	DNA replication	RnrL(ribonucleoside-diphosphate reductase large subunit)	1.0(2.4e-012)	0.8(4.10e-08)	–	0.6(0.000003)	0.6(0.000005)
7	pro-apoptotic (predicted)	CG5059(–)	1.0(2.0e-011)	1.3(6.1e-012)	-0.5(0.003960)	–	0.5(0.000213)
8	aka ‘Dark’; pro-apoptotic	Ark(Apaf-1 related killer)	1.1(9.3e-009)	0.6(0.000424)	–	0.3(0.049208)	0.4(0.009484)
9	RNA interference, cell death	AGO2(Argonaute 2)	1.3(1.2e-011)	0.8(1.7e-008)	–	–	–
10	aka ‘Dronc’; pro-apoptotic	Nc(Nedd2-like caspase)	1.6(4.6e-011)	0.9(2.0e-007)	–	0.3(0.019869)	–
11	pro-apoptotic	p53(p53-like regulator of apoptosis and cell cycle)	1.7(3.4e-013)	1.4(5.4e-009)	–	–	–
12	predicted VEGF receptor binding	Pvf2(VEGF-related factor 2)	1.9(2.3e-013)	2.2(6.1e-011)	–	0.4(0.003801)	0.4(0.001485)
13	predicted inhibitor of apoptosis	CG7188(–)	2.0(8.6e-009)	1.2(1.6e-010)	0.3(0.036872)	–	–
14	JNK signaling	puc(puckered)	2.2(3.2e-014)	1.2(2.0e-008)	–	0.3(0.040766)	0.6(0.000583)
15	autophagic cell death	Mmp1(Matrix metalloproteinase1)	2.5(2.6e-012)	3.6(0)	−0.4(0.023325)	0.5(0.000006)	0.9(8.8e-008)
16	**pro-apoptotic**	**skl(sickle)**	**2.6(2.2e-007)**	–	–	**0.2(0.006202)**	**0.2(0.006266)**
17	**pro-apoptotic**	**W(hid/W)**	**2.8(0)**	**1.4(1.1e-010)**	**-0.7(0.000616)**	**–**	**0.7(0.000014)**
18	**pro-apoptotic**	**rpr(reaper)**	**3.1(0)**	**1.5(6.8e-010)**	**0.5(0.002109)**	**0.5(0.001486)**	**0.4(0.011275)**
19	JNK signaling	Traf4(TNF Receptor Associated Factor1)	4.0(0)	2.4(5.2e-012)	–	1.0(0.000003)	0.7(0.000104)
20	JNK signaling	egr(Eiger)	5.8(0)	3.7(3.0e-013)	0.3(0.034627)	1.3(0.000399)	0.7(0.012767)
21	autophagic cell death	LysS(Lysozyme S)	5.6(2.4e-010)	−3.3(0.000003)	2.6(0.001140)	−0.8(0.010742)	−2.8(0.000433)
22	pro-apoptotic	Corp(Companion of reaper)	6.2(0)	5.0(1.1e-015)	–	0.7(0.015186)	0.7(0.010899)

The values shown are log_2_. The cut-off values were 1.5 fold or more (log_2_ of 0.585 or greater) with p<0.005 compared to un-irradiated controls, at 2 hr or 18 hr after irradiation or both. ‘–’ = the gene was not significantly induced with respect to neither p-value or fold change. p-value of 0 means p<1e-10. y = *yw* control; p = *p53* mutants, ‘−’ = −IR (0 R); ‘+’ = +IR (4000 R); 2 = 2 hr after irradiation, 18 = 18 hr after irradiation. If there is data for more than one probe set is available for a gene, the set with the best p value was considered.

### The effect of IR on ‘DNA-damage response’ genes

The ‘DNA damage response’ category consists of 37 induced and 53 repressed genes (Table 3, ±1.5-fold or greater, p<0.005). As expected, genes with roles in DNA repair, recombination and by-pass synthesis (in italics) are over-represented among the induced (top half of Table 3). In contrast, repressed genes (bottom half of Table 3) include those encoding essential replication factors (in bold font). Among the latter are genes encoding the components of the Pre-Replication Complex: subunits of the Origin Recognition Complex, ORC5 and ORC6; MCM3, MCM5, MCM6 and MCM7; positive and negative regulators of MCM loading, Cdc6, Dup and Geminin; and CDC45 that recruit DNA polymerases to the pre-RC; three subunits of DNA polymerase α; sliding clamp, PCNA; and, clamp loader RFC. Significant repression of these genes remains at 18 hr after irradiation. A systematic repression of DNA replication genes by IR has not been reported before. It is possible that such genes may have been placed into ‘cell cycle’ or ‘cell proliferation’ clusters in other analyses.

**Table 3:** DNA damage response genes induced or repressed in *yw* (±1.5-fold or greater change, p<0.005).

	other information (Flybase)	gene	fold change(p)			
			y2− vs y2+	y18− vs y18+	p2− vs p2+	p18− vs p18+
**INDUCED at 2 or 18 hr or both**						
1	*DNA repair*	*agt(O-6-alkylguanine-DNA alkyltransferase)*	3.8(0)	3.6(1.1e-016)	1.2(4.9e-007)	2.0(3.7e-010)
2	RNA-dependent DNA polymerase (predicted)	CHKov1(CHKov1)	3.2(2.0e-010)	5.5(2.9e-013)	–	–
3	*DNA repair*	*Ku80(Ku80)*	2.9(1.1e-016)	2.0(7.5e-012)	1.2(0.000009)	1.1(6.1e-007)
4	*associate with Ku70/80 complex*	*Irbp(Yolk protein factor 1b)*	2.6(8.9e-015)	2.1(1.2e-012)	1.1(0.000012)	1.0(0.000048)
5	*translesion synthesis*	*mus205(mutagen-sensitive 205)*	2.6(4.4e-016)	1.7(1.8e-011)	0.4(0.014217)	0.8(0.000004)
6	*DNA repair*	*Lig4(ligase4)*	2.6(3.5e-013)	1.9(4.2e-007)	1.0(0.008889)	0.6(0.029897)
7	*DNA repair, replication*	*lig3(DNA ligase III)*	2.4(1.8e-013)	0.7(0.001965)	–	0.6(0.001044)
8	*DNA damage signaling*	*rad50(rad50)*	2.3(1.1e-014)	2.0(5.8e-013)	0.5(0.001039)	0.6(0.000013)
9		CG6171(Anon-becker2)	2.1(1.1e-014)	1.7(6.2e-011)	0.7(0.000172)	0.6(0.000004)
10	DNA binding (ecdyson biosynthesis)	kay(shroud)	1.9(1.5e-013)	0.7(0.000316)	−0.5(0.002455)	0.3(0.029236)
11	Jun-related antigen, JNK signaling	Jra(Jun oncogene)	1.8(7.9e-012)	0.7(0.000021)	–	–
12	*DNA repair*	*mus210(xeroderma pigmentosum group C complementing factor)*	1.8(3.7e-014)	0.9(6.3e-009)	–	0.3(0.006671)
13	*translesion synthesis*	*DNApol-eta(DNApol-eta)*	1.7(4.0e-012)	0.9(0.000008)	–	–
14	*DNA damage signaling*	*mre11(meiotic recombination 11)*	1.7(3.6e-014)	1.3(5.8e-012)	0.6(0.000151)	0.9(0.000003)
15	*DNA repair*	*mei-9(meiotic 9)*	1.7(1.4e-009)	0.9(0.000079)	0.5(0.044112)	0.5(0.019780)
16	**DNA replication**, *repair*	RpA-70(Drosophila Replication Protein A)	1.5(4.0e-014)	1.4(6.9e-010)	–	0.5(0.000022)
17	development, signaling	Btk29A(Btk family kinase at 29A)	1.5(2.0e-012)	0.8(0.000007)	−0.3(0.046417)	–
18	Elongation Factor 2 kinase	PEK(PEK)	1.4(2.6e-013)	1.0(3.5e-008)	–	0.4(0.002521)
19	*DNA repair*	*XRCC1(XRCC1)*	1.3(4.2e-008)	1.5(4.9e-007)	–	–
20	exonuclease (predicted)	CG12877(–)	1.2(8.9e-009)	–	0.4(0.015037)	0.4(0.044350)
21	multiple roles including *DNA repair*	*UbcD6(Ubiquitin conjugating enzyme)*	1.2(1.6e-010)	1.3(5.8e-010)	0.5(0.030280)	0.2(0.044904)
22	predicted hydrolase, cell polarity	gkt(glaikit)	1.0(0.001345)	1.4(0.000006)	–	–
23	*translesion synthesis*	*DNApol-iota(DNApol-iota)*	1.0(0.000418)	0.9(0.006934)	–	–
24	**DNA replication** and *repair*	RnrL(ribonucleoside-diphosphate reductase large subunit)	0.96(2.4e-012)	0.75(4.1e-08)	−0.07(0.58986)	0.58(0.000003)
25	*DNA repair (predicted)*	*CG5524(*–*)*	0.9(0.000012)	–	–	–
26	checkpoint	pic(piccolo)	0.8(6.0e-009)	0.8(2.5e-008)	–	–
27	*DNA repair*	*spn-A(Spindle-A)*	0.8(0.000072)	0.6(0.000648)	–	–
28	Src kinase homolog	Src42A(Suppressor of pole hole)	0.7(3.1e-008)	0.5(0.000314)	–	–
29	chromatin regulation	Ssrp(structure-specific recognition protein)	0.7(2.4e-007)	0.8(1.4e-007)	–	–
30	guanylate kinase (predicted)	pyd(tamou)	0.7(0.000003)	–	–	–
31	checkpoint	RfC4(Replication factor C subunit 4)	0.6(1.2e-008)	0.7(1.2e-007)	–	0.5(0.000363)
32	recombination	c(3)G(crossover suppressor on 3 of Gowan)	0.6(0.022482)	0.8(0.001269)	–	–
33	**DNA replication**	**EndoG(CG8862)**	0.6(0.000367)	0.4(0.044169)	–	–
34	cell cycle regulation	Rbf(Retinoblastoma-family protein)	0.6(0.000064)	–	–	–
35	*DNA repair*	*Ercc1(Ercc1)*	0.4(0.001146)	0.6(0.000128)	–	–
36	**DNA replication (predicted)**	**CG15220(–)**	0.3(0.001870)	1.0(2.3e-009)	–	0.5(0.000034)
37	DNA metabolism	Top1(topoisomerase I)	–	0.6(0.000572)	–	–
**REPRESSED at 2 or 18 hr or both**						
1	replication fork protection (predicted)	CG10336(–)	−2.0(1.6e-008)	−0.6(0.000565)	–	–
2	Rnase H (predicted)	CG13690(–)	−1.9(8.0e-008)	−0.8(0.001579)	−0.5(0.004728)	0.3(0.026737)
3	**DNA replication**	**Orc6(Origin recognition complex subunit 6)**	−1.8(8.1e-009)	−1.0(0.000011)	–	0.4(0.006813)
4	**DNA replication**	**DNApol-alpha60(“DNA polymerase alpha 58,000 beta subunit”)**	−1.8(2.0e-008)	−0.5(0.020794)	−0.5(0.019469)	0.3(0.035621)
5	**DNA replication**	**Pole2(Pole2)**	−1.7(4.1e-008)	−0.6(0.001494)	−0.5(0.005458)	0.4(0.005395)
6		Hsp70Bc(heat shock 70)	−1.7(0.034517)	−0.8(0.223137)	0.9(0.382707)	−2.1(0.000112)
7	**DNA replication**	**dup(Double-parked)**	−1.5(2.1e-010)	–	−0.5(0.007475)	0.6(0.000013)
8	*DNA repair*	*RecQ4(RecQ4)*	−1.5(0.000284)	−0.6(0.032397)	–	0.5(0.019571)
9	**DNA replication**	**RfC3(Drosophila replication factor C)**	−1.4(7.5e-009)	−0.3(0.020703)	−0.5(0.005448)	0.6(0.000044)
10	**DNA replication**	**Cdc6(Cdc6)**	−1.4(0.000006)	–	–	–
11	**DNA replication**	**DNApol-alpha73(DNA polymerase 73K)**	−1.4(0.000002)	–	–	0.4(0.022580)
12	helicase (predicted)	CG5924(d-mtDNA helicase)	−1.2(0.000194)	–	–	–
13	**DNA amplification**	**hd(humpty dumpty)**	−1.2(0.000022)	−0.4(0.022023)	−0.6(0.007042)	–
14	**DNA replication**	**DNApol-epsilon(DNA polymerase epsilon)**	−1.2(0.000019)	–	–	–
15		cutlet(gilead)	−1.1(0.000017)	−0.7(0.006384)	–	–
16	chromatin silencing, **DNA replication**	**Mcm10(Sensitized chromosome inheritance modifier 19)**	−1.1(0.000004)	–	−0.5(0.004123)	0.4(0.004897)
17	**DNA replication**	**Mcm3(Minichromosome maintenance 3)**	−1.0(2.2e-010)	−0.5(0.000038)	−0.3(0.010981)	0.3(0.034050)
18	**DNA replication**	**DNApol-alpha180(DNA polymerase 180K)**	−1.0(0.000363)	–	–	0.4(0.013874)
19	Src kinase homolog	Src64B(Src oncogene at 64B)	−1.0(0.000013)	−1.0(0.000508)	–	–
20	3′-5′ exonuclease (predicted)	WRNexo(CG7670)	−0.9(3.0e-008)	−0.8(0.000117)	−0.3(0.021623)	–
21	**DNA replication**	**Mcm5(Minichromosome maintenance 5)**	−0.9(1.3e-009)	−0.5(0.000126)	–	0.3(0.014684)
22	nuclease (predicted)	mms4(CG12936)	−0.9(0.000052)	−0.6(0.008131)	–	0.4(0.032641)
23	**helicase, DNA replication**	**Psf2(Psf2)**	−0.9(0.000008)	–	–	–
24	**DNA replication**	**CDC45L(Transcription unit D)**	−0.8(2.6e-007)	−0.4(0.009211)	−0.3(0.038574)	0.4(0.005622)
25	microtubule binding, chromosome segregation	Klp3A(Kinesin-Like-Protein-at-3A)	−0.8(2.6e-007)	−0.5(0.000180)	−0.3(0.047748)	–
26	meiotic recombination	trem(CG4413)	−0.8(0.000137)	–	–	–
27	*DNA repair*	*mu2(mutator 2)*	−0.8(0.000018)	−0.6(0.000528)	–	0.4(0.012942)
28	mitotic spindle, transcription	mip130(Myb-interacting protein 130)	−0.8(0.000015)	–	–	−0.3(0.019180)
29	cell cycle regulation, cell death, transcription	E2f2(E2F transcription factor 2)	−0.8(0.000006)	−0.5(0.000132)	–	–
30	Ubiquitin ligase (predicted)	ago(archipelago)	−0.7(8.1e-007)	−0.5(0.000194)	−0.2(0.045051)	–
31	checkpoint	grp(grapes)	−0.7(2.7e-007)	−0.5(0.000245)	−0.7(0.046548)	–
32	**DNA replication**	**geminin(geminin)**	−0.7(2.1e-007)	−0.5(0.000010)	–	–
33	cell cycle regulation	pim(pimples)	−0.7(1.1e-007)	−0.7(0.000002)	–	–
34	*DNA mismatch repair*	*Mlh1(Mlh1)*	−0.7(0.001590)	−0.4(0.033836)	–	–
35	*DNA repair (predicted)*	*Fen1(Flap endonuclease 1)*	−0.7(0.000075)	−0.3(0.021554)	–	–
36	**DNA replication**	**Orc5(lethal(2)34Df)**	−0.7(0.000057)	−0.4(0.029409)	–	–
37	**DNA replication**	**Gnf1(germline transcription factor)**	−0.7(0.000003)	−0.3(0.023688)	–	0.2(0.048634)
38	**DNA replication**	**Mcm6(Minichromosome maintenance 6)**	−0.7(0.000001)	−0.5(0.000029)	–	–
39	**DNA replication**	**mus209(proliferating cell nuclear antigen)**	−0.6(9.9e-007)	–	–	0.5(0.000024)
40	DNA binding, segment specification	crm(swollen-antenna)	−0.6(0.013153)	−0.9(0.000257)	–	–
41	DNA transposition (predicted)	CG4570(–)	−0.6(0.002225)	–	–	–
42	transcription regulation	mip120(Myb-interacting protein 120)	−0.6(0.000151)	−0.8(0.000041)	–	−0.3(0.009726)
43	*DNA repair*	*Mms19(Mms19)*	−0.6(0.000027)	–	–	–
44	microtubule organization	CG8142(–)	−0.6(0.000023)	–	–	0.3(0.001450)
45	microtubule binding, chromosome segregation	nod(no distributive disjunction)	−0.5(0.000143)	−0.6(0.000073)	–	–
46	**DNA replication**	**Mcm7(Minichromosome maintenance 7)**	−0.5(0.000040)	−0.7(0.000002)	–	–
47	transcription regulation	woc(without children)	−0.4(0.000022)	−0.6(0.000006)	–	–
48	post-embryonic development	vg(vestigial)	−0.3(0.013039)	−1.0(7.9e-009)	–	–
49	**chorion gene amplification**	**chif(chiffon)**	−0.2(0.022017)	−0.8(6.1e-008)	–	–
50	**DNA replication** and *repair*	RnrS(ribonucleoside-diphosphate reductase small subunit)	−1.0(1.00e-10)	−0.8(1.2e-08)	−0.6(0.000188)	0.4(0.000186)
51	response to hydrogen peroxide	Cat(catalase)	–	−0.7(0.000090)	–	−0.3(0.029640)
52	transcription initiation	Ssl1(Ssl1)	–	−0.8(7.8e-007)	–	–

The values shown are log_2_. The cut-off values were 1.5 fold or more (log_2_ of 0.585 or greater) with p<0.005 compared to un-irradiated controls, at 2 hr or 18 hr after irradiation or both. ‘–’ = the gene was not significantly induced with respect to neither p-value or fold change. p-value of 0 means p<1e-10. y = *yw* control; p = *p53* mutants, ‘−’ = −IR (0 R); ‘+’ = +IR (4000 R); 2 = 2 hr after irradiation, 18 = 18 hr after irradiation. If there is data for more than one probe set is available for a gene, the set with the best p value was considered.

### Gene expression changes in irradiated p53 mutants

As described in a preceding section, Table S1 lists all genes that show a ±1.5-fold or greater change (p<0.005) between ANY −IR/+IR sample pair at 2 or 18 hr post irradiation, in *yw* or *p53* discs. Using these criteria, the numbers of genes that show altered expression after irradiation in *yw* discs were 1257 and 1315 respectively at 2 and 18 hr after IR (Figure S4). The corresponding numbers for *p53* mutants were 284 and 229, at 2 and 18 hr respectively. In other words, loss of p53 results in ∼5-fold reduction in the number of genes that respond to IR. In addition, even for genes whose expression changed significantly in *p53* mutants, nearly all show a dampened response compared to *yw* controls (e.g. RnrL and Corp in Table 2). Interestingly, the dependency on p53 is not limited to genes that are induced by IR; several genes that show absent or dampened response in *p53* mutants are genes that are repressed by IR in *yw* discs. We note in particular genes in the ‘DNA damage response’ category that are repressed by IR in *yw* discs. These are not repressed to the same degree in *p53* mutant discs (Table 3). Specifically, most DNA replication genes described in the preceding section are repressed in *p53* mutants but to a lesser degree. This suggests the existence of a p53-dependent mechanism to repress DNA replication genes after irradiation as well as a weaker p53-independent mechanism. Similarly, genes induced in *yw* discs are either not induced or induced to a lesser degree in *p53* mutants. These data likewise suggest the existence of p53-dependent and p53-indepedent mechanisms that cooperate to activate DNA repair, cell death and other genes after irradiation. In addition, genes that function in wing disc development show expression changes in *yw* discs but are notably absent in *p53* (Figure S4). This is in agreement with a recent report that another function of *p53* is to delay development in response to IR in larvae, thereby coordinating cellular responses with the developmental program [19].

### Candidates for regulators of p53-independent apoptosis

**Figure 1:**
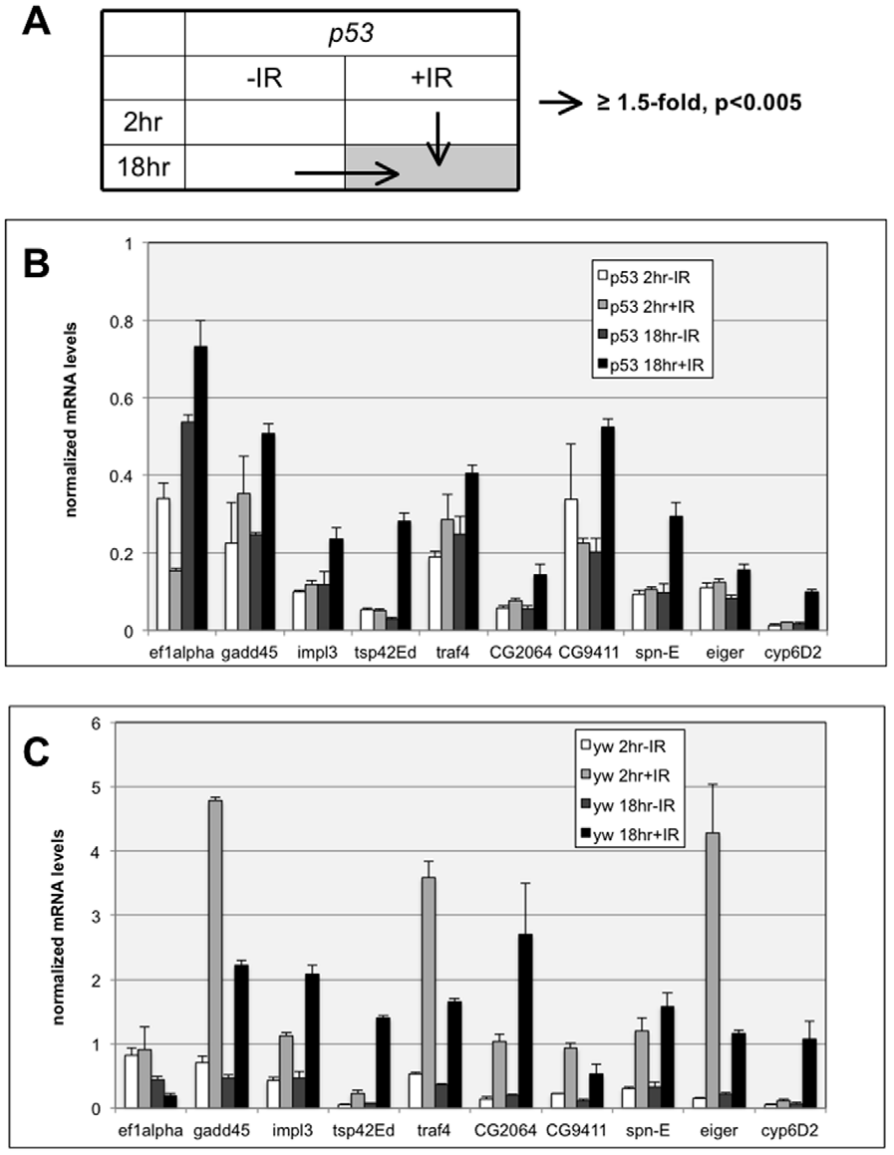
Validation by quantitative RT-PCR of 10 candidate genes identified in microarray analysis. Total RNA was isolated from wing imaginal discs of third instar larvae at 2 or 18 hr after exposure to 0 or 4000R of X-rays. RNA was reverse-transcribed into cDNA and subjected for quantitative RT-PCR analysis as described in Methods. a-tubulin levels were used to normalize the graphs. Error bar = 1 STD. (A) A schematic representation of data comparisons used to select candidates for validation. The gray box denotes the time at which apoptosis becomes detectable in *p53* mutants. (B) Q-RT-PCR results in *p53* mutant wing imaginal discs (B) Q-RT-PCR results in wild type (*yw*) wing imaginal discs.

Generally speaking, p53-dependent mechanisms that respond to DNA damage are better characterized than p53-independent mechanisms. In order to better understand p53-independent mechanisms, we identified genes that show a response profile similar to that of Drosophila pro-apoptotic Smac/DIABLO orthologs. At 2 hr after irradiation, *rpr*, *hid* and *skl*, are induced in *yw* discs but are either not induced or induced to a lesser degree in *p53* mutant discs (Table 2, bold). At this time point, *yw* discs are about to undergo apoptosis but *p53* mutant discs are not. At 18 hr after irradiation, when p53-independent apoptosis occurs, these genes are induced in *p53* mutants relative either to age-matched non-irradiated controls (p18− vs. p18+ in Table 2) or to *p53* mutants at 2 hr after irradiation (p2+ vs. p18+ in Table 2). Therefore, we identified genes whose expression in *p53* mutants (1) increased significantly at 18 hr after irradiation compared to un-irradiated *p53* mutants, and (2) showed a significant increase in IR+18 hr *p53* mutant compared to IR+2 hr *p53* mutant (≥1.5-fold, p<0.005; arrows in Figure 1A). Of ∼13,000 genes analyzed, 87 fulfilled these criteria (Table S4). None of these were induced in 18 hr-IR samples compared to 2 hr-IR samples using similar cut-offs; that is, induction in 18 hr+IR samples relative to 2 hr+IR samples is not due to aging of larvae. Of the 87 genes, 7 genes were also induced by IR at 2 hr after irradiation in *p53* mutants (p2+ vs. p2−, ≥1.5-fold, p<0.005, in bold in Table S4). The level of induction, however, was less than that at 18 hr+IR such that p18+IR level was significantly higher than p2+IR level (fulfilling criteria #2). We reasoned that these genes may be induced in a p53-independent manner at 2 hr after irradiation but their levels climbed higher at longer times, and thus could contribute to the delayed apoptotic response.

### Confirmation of gene expression changes by Q-RT-PCR

Nine genes from Table S4 (italicized) were selected for further analysis (Table 4). These span the whole range of fold-inductions and p-values seen at 18 hr in *p53* mutants. In addition, *eiger* was chosen as the 10^th^ gene to confirm by Q-RT-PCR. *eiger* was significantly induced at 18 hr after irradiation (p18+ samples) compared to age-matched un-irradiated controls (p18- samples), but not compared to 2 hr+IR samples. There are two reasons behind our interest in *eiger*, which encodes a TNF superfamily ligand that activates the Drosophila JNK pathway. First, Eiger and JNK were shown previously to positively modulate p53-independent apoptosis [9]. Second, we found two other mediators of JNK signaling, Traf4 and GADD45, were induced at 18 hr in irradiated *p53* discs. Traf4 encodes Drosophila TNF Receptor Associated Factor 1, which is required for JNK signaling [21]. GADD45 homologs in mammals mediate JNK activation in response to stress [22], and Drosophila GADD45 shows genetic interaction with JNK pathway components in egg development [23]. Because of apparent relevance of JNK pathway to p53-independent apoptosis, we included *eiger* among candidates to confirm by Q-RT-PCR. We find that Q-RT-PCR confirmed the profile of expression changes after irradiation in p53 mutants seen in microarray-based analysis for all ten candidates (Figure 1B), although fold-change may differ between microarray and Q-RT-PCR for some genes. Specifically, for each gene, transcript levels in 18 hr+IR samples were significantly higher than in 18 hr−IR or 2 hr+IR samples in Q-RT-PCR analysis, which is in agreement with the microarray data. The transcript level changes for corresponding samples in *yw* discs also are in agreement with microarray results (Figure 1C, Table 4).

**Table 4:** Ten candidate genes for confirmation by Q-RT-PCR.

	Additional information (Flybase)	Gene	fold change (p value)				
			y2− vs y2+	y18− vs y18+	p2− vs p2+	p18− vs p18+	p2+ vs p18+
1	translation elongation	Ef1alpha100E(elongation factor 1-alpha F2)	–	–	−2.0(4.8e-007)	1.2(3.0e-008)	3.0(1.0e-011)
2	unknown function	Tsp42Ed(tetraspanin 42E)	3.4(7.0e-009)	6.2(0)	–	2.8(4.5e-007)	2.8(4.5e-007)
3	electron carrier, oxidation-reduction	Cyp6d2(Cyp6d2)	1.8(0.000032)	5.9(7.0e-015)	–	2.2(0.000001)	2.1(0.000004)
4	RNA helicase	spn-E(Spindle-E (homeless))	2.7(1.8e-010)	3.5(2.1e-011)	–	2.3(1.4e-007)	2.0(0.000303)
5	unknown function	CG9411(–)	1.8(0.000017)	3.6(1.2e-008)	–	2.1(2.7e-007)	1.8(0.000003)
6	unknown function	CG2064(–)	3.8(4.8e-014)	4.8(0)	–	1.9(1.0e-009)	1.4(0.000007)
7	L-lactate dehydrogenase (predicted)	ImpL3(lactic DH)	2.0(8.8e-011)	4.1(2.2e-014)	–	1.2(0.000293)	1.2(0.001270)
8	JNK cascade	Gadd45(Gadd45)	4.6(4.4e-016)	3.0(1.0e-010)	1.3(0.000593)	1.6(0.000013)	1.1(0.000288)
9	JNK cascade	egr(Eiger)	5.8(0)	3.7(3.0e-013)	0.3(0.034627)	1.3(0.000399)	0.7(0.012767)
10	JNK cascade	Traf4(TNF Receptor Associated Factor 1)	4.0(0)	2.4(5.2e-012)	–	1.0(0.000003)	0.7(0.000104)

The genes are shown with functional information extracted from Flybase. Expression changes (log2 of fold change) for 5 pair-wise comparisons are also given.‘–’ = the gene was not significantly induced with respect to neither p-value or fold change. p-value of 0 means p<1e-10. y = *yw* control; p = *p53* mutants, ‘−’ = −IR (0 R); ‘+’ = +IR (4000 R); 2 = 2 hr after irradiation, 18 = 18 hr after irradiation. If there is data for more than one probe set is available for a gene, the set with the best p value was considered.

### The role of EF1-a 100E in p53-independent apoptosis

Of the ten candidates whose induction in *p53* mutants was confirmed by Q-RT-PCR, we chose to analyze EF1-a 100E further. EF1-a 100E encodes an essential translation elongation factor and was chosen for three reasons. First, in microarray analysis, it showed the greatest level of induction from 2 hr to 18 hr after irradiation in *p53* discs (8-fold, Table 4, last column). Part of the reason is that EF1-a 100E is actually repressed at 2 hr after IR in p53 mutants. In *yw* controls, EF1-a 100E is repressed at both 2 and 18 hr (Table S1), but p values were too high for inclusion in Table 4. Second, a reduction in protein synthesis capacity of the cell has been proposed to target the cell for p53-independent apoptosis ([9] and (reviewed in [24]). Specifically, it was proposed that IR-induced chromosomal breaks result in the loss of loci that encode ribosomal proteins, which are scattered through the genome. Consequent reduction in protein synthesis and growth renders the cell a ‘looser’ relative to neighboring cells. Cell competition is known to induce apoptosis in loser cells [25], [26]. Third, induction of EF1-a expression by IR is conserved in human cells; a previous microarray analysis showed the REPRESSION of both EEF1A1 and EEF1A2, encoding EF1-a homologs, in human fibroblasts at 2 hr after irradiation [27]. This is what we see in *yw* discs at 18 hr after IR and *p53* discs at 2 hr after IR. Thus, we sought to investigate whether the induction of EF1-a we see at 18 hr in p53 mutants has any significance.

**Figure 2:**
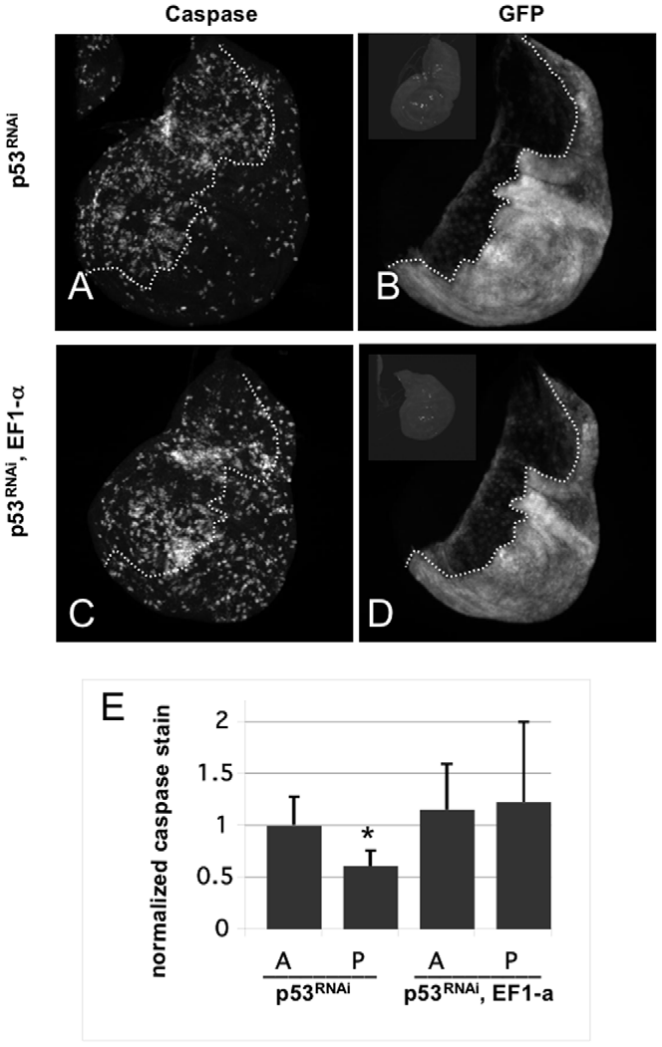
EF1a mutants show elevated levels of IR-induced apoptosis in a p53-depleted background. Wing imaginal discs were dissected from 3^rd^ instar larvae at 24 hr after exposure to 0 (−IR) or 4000R (+IR) of X-rays. Apoptosis was detected by staining with an antibody to active cleaved Caspase 3. GFP boundary is used to mark the boundary between anterior and posterior compartments. *en-GAL4* is active only in the posterior compartment. (A and B) p53^RNAi^ = *en-GAL4>UAS-dsRNA* against p53, *UAS-GFP*. Caspase stain is in (A) and GFP fluorescence is in (B). (C and D) p53^RNAi^, EF1-a = same as in (A) but in homozygous EF1-a mutant background. (Insets in B and D) show unirradiated control discs stained for caspase, to show little or no apoptosis in the absence of irradiation. The insets are shown with increased brightness to make disc outlines discernable. (E) Mean caspase signal in each compartment is quantified and shown normalized to the mean caspase signal of the anterior (A) compartment in p53^RNAi^ discs (the first bar). Caspase signal in the posterior (P) compartment of the same discs are reduced significantly compared to the A compartment (p<0.001, two-tailed t-test). This is expected; the level of p53-independent apoptosis is about half of p53-dependent apoptosis at 24 hr after irradiation [8]. Caspase signal in the A compartment of ‘p53^RNAi^, EF1-a’ discs are not significantly different from the caspase signal in the A compartment of p53^RNAi^ discs (p = 0.29), suggesting that reduction of EF1-a alone did not affect the level of apoptosis when p53 is present. Caspase signal in the A and P compartments of ‘p53^RNAi^, EF1-a’ discs are not significantly different from (p = 0.70). Caspase signal in P compartment of ‘p53^RNAi^, EF1-a’ discs are significantly greater than the signal in the P compartment of p53^RNAi^ discs (p<0.05). The data are from 12 p53^RNAi^ discs and 22 p53^RNAi^, EF1-a discs in two different experiments. Error bar = 1 STD.

EF1-a 100E is an essential gene; null alleles are lethal. Therefore, we asked if a hypomorphic mutation in EF1-a 100E has any consequence on apoptosis, in the presence and absence of p53. To deplete p53 by RNAi, double-stranded RNA against p53 was driven in the posterior (P) compartment of wing disc using *engrailed*-GAL4. Cells in the anterior (A) compartment contain the same transgenes and mutations but do not express GAL4, and therefore serve as control. We find that depletion of p53 by RNAi results in delayed and reduced apoptosis in the P compartment compared to the A compartment (Figure 2 and data not shown). At 24 hr after irradiation, apoptosis in the p53-depleted half is about 50% of controls, in agreement with published data using p53 null mutants [8]. With p53 (RNAi) in the EF1-a 100E mutant background, the two halves have the about the same level of apoptosis and is similar to that of the A compartment in p53^RNAi^ only controls. Thus, reduction of EF1-a 100E levels elevated p53-independent apoptosis. We confirmed these results using trans-heterozygotes of the same allele of EF1-a 100E and a chromosomal deficiency that removes the gene (Figure S5). We conclude that EF1-a 100E normally inhibits p53-independent apoptosis.

## Discussion

We are aware of 4 published studies on genome-wide gene expression changes in response to ionizing radiation (IR) in Drosophila [17], [28], [29], [30]. All used embryos and applied various cut-offs for fold-change and p-values. In the first published study, 17 of ∼13,000 genes were induced 1.7 fold or higher at 15–240 min after exposure to 4000 R of X-rays, translating to 0.1% hit rate [17]. The second study found 105 genes of ∼5500 examined induced at least 2-fold (p<0.05) at 3 hr after exposure to 4000R, translating to a ∼2% hit rate [29]. The third study reports a 1.2–3.0% hit rate using a 1.6-fold cut-off at 90 min after exposure to 4000 R of X-rays in embryos [28]. The fourth study reported only the ‘top 50’ genes, so we could not compute a hit rate [30]. Our hit rate of 2.8% (2-fold or higher induction at p<0.001) is therefore close to what was seen in two previous studies, but could be higher with less stringent cut-offs.

Our hit rate in wing imaginal discs is comparable to what has been reported for irradiated IMR-90 human fibroblasts; 1164/∼41,000 transcripts (2.8%) were induced at 2 hr after exposure to 1 Gy (100R) of g-radiation [27]. Although the radiation doses used are different (4000R vs. 100R), LD50s are also proportionately different in Drosophila larvae and cultured human fibroblasts (4000R vs. 300–400 R) [18], [31]. Gene ontology groups among IR-induced genes in Drosophila larvae (Table 1, this study) and human fibroblasts [27] overlap for the expected groups such as ‘DNA repair’ and ‘apoptosis’, but also include a less-expected group of genes involved in coenzyme biosynthesis/metabolism. This coincidence is good given that different cell types from the same organism can react very differently to ionizing radiation; genome-wide analyses show that genes induced by IR in cultured human embryonic stem cells and cultured human fibroblasts overlap by just two genes [27], [32].

The dataset we report here for *Drosophila* encompasses two time points to address both p53-dependent (early) responses and p53-independent (late) apoptotic responses in response to ionizing radiation. We are not aware of a comparable dataset in the literature to perform a direct comparison. Nonetheless, there are numerous studies on the role of p53 in cellular stress including ionizing radiation. A recent comparison of the datasets on 5 different human cell lines, both malignant and non-malignant, found that even with the same stress agent, the vast majority of changes (>90% of genes) were cell line-specific [33]. For instance, only 54 genes were induced by IR in at least 3 of 5 cell lines in these studies. Nonetheless, we see many parallels to what we find in Drosophila. First, as many genes were repressed as were activated by IR. Second, p53 was the key mediator of these changes. Third, IR-induced core group (common among cell lines and conditions) includes GO categories for regulation of apoptosis, regulation of the cell cycle, response to stress, DNA damage response and signal transduction. These categories are also present among the IR-induced groups in Drosophila (Table 1). While there is good correspondence for functional categories between *Drosophila* and human datasets, the actual identities of genes differed significantly. Of the 54 human genes induced by IR in at least 3 of 5 cell lines, only 3 had clear sequence and functional homologs in *Drosophila* that were also induced by IR: GADD54, REV3 (mus205 in *Drosophila*) and POLH/pol-eta.

Because our hit rate is most similar to what was reported by Akdemir et al. [28] among previous studies in Drosophila embryos, we compared our genes to theirs. The published study identified 29 “high stringency IR-induced genes”, 18 of which were also found by us at 2 hr after irradiation (Table 2). These are CD6272, escl, mre11, eIF6, CG17836, CG12171, CG18596, CG11897, CG6171, rpr, corp, skl, hid, egr, CG9836, mus205, CG5664 and mus210. In addition, our list includes GstD4, GstE3, GstE5, GstE6 and GstE7 whereas the published list includes GstD5; all are enzymes in the synthetic pathway for the antioxidant glutathione. Thus, there is general agreement between the data sets. Other differences could be technical or due to differences in how embryos and larval wing imaginal discs respond to IR. It is known that even within the larva, different tissues respond differently to genotoxic stress (for example [15], [34]). We will need a similar data set from another larval tissue, however, to address tissue-specificity in radiation response in Drosophila.

Our findings extend to the larvae a conclusion based on gene expression analysis in embryo, that p53 is the major regulator of IR-induced changes in the transcriptome. The number of genes affected by IR in p53 mutants was less than one fifth of what is seen in *yw* (Table S1, Figure S4). Furthermore, not only were the numbers smaller but also the degree of change, whether induction of repression, were smaller in *p53* mutants.

A new theme that emerged from our data is that induction of apoptosis accompanies changes not only in pro-apoptosis genes but also in anti-apoptosis genes. For example, E2F2 was found previously to inhibit p53-independent apoptosis [10]. E2F2 was repressed by IR at both 2 and 18 hr in *yw* discs (Table S1), and this repression was dependent on p53. This raises the possibility that repression of E2F2 by p53 contributes to optimal induction of apoptosis. In p53 mutants, E2F2 is not repressed to the same extent and act to inhibit apoptosis. This is consistent with our previous findings that double mutants of p53 E2F2 show more apoptosis than p53 single mutants [10].

During normal cell cycle progression, E2F1 activates and E2F2 represses genes encoding essential replication factors at the G1/S boundary, in preparation for DNA synthesis [35]. Interestingly, we find that many of these genes were also repressed by IR. Because E2F2 was repressed by IR as well, repression of S phase genes is unlikely to be a consequence of reduced E2F2. Instead, we find that p53 is required for optimal repression of S phase genes after irradiation. These results suggest the existence of a transcriptional regulatory module to repress S phase genes that function independently of E2F2 but may involve p53. More work will be needed to determine if such a module exists and what role p53 plays in it.

Eiger, a ligand that activates of JNK signaling, is dispensable for p53-dependent apoptosis [17]. JNK signaling, however, was found to promote p53-independent apoptosis [9]. Because Eiger is induced at 2 hr after irradiation in p53 mutants, when p53-independent apoptosis is yet to be initiated, induction of *eiger* cannot be sufficient for apoptosis in p53 mutants. We find that two other regulators of JNK signaling, Traf4 and GADD45, are induced at 18 hr after irradiation compared to 2 hr after irradiation in p53 mutants (Table S3). Possibly, additional induction of Traf4 and GADD45 corporate with Eiger to increase the JNK signal and thereby promote apoptosis at later time points after irradiation in p53 mutants. Indeed, this is yet another theme that has emerged from this work and others, that there is not a single pathway to p53-independent apoptosis but that several gene products contribute. Some such as JNK and E2F1 promote p53-independent apoptosis while others such as E2F2 and EF1–a100E repress it. Induction of these genes by IR is not only delayed but also dampened in p53 mutants (compare y-axes in Figures 1B and C). Low level of induction may be why contribution from several gene products is needed to induce apoptosis in *p53* mutants.

EF1–a100E is induced by IR but only in p53 mutants at 18 hr post irradiation. Single mutants in EF1–a100E show a normal apoptotic response (“A” compartment in Figure 2B), but the caveat is that only partial loss-of-function alleles can be used to study this essential gene. Nonetheless, reduction of EF1–a100E increased p53-independent apoptosis, suggesting that EF1–a100E is either neutral (in *yw* background) or anti-apoptotic (in *p53*-reduced background).

How might EF1–a be anti-apoptotic/pro-survival? We can envision at least three possible scenarios. First, irradiation is known to change the profile of mRNAs on the ribosome in mammalian cells [36]. A similar analysis has not been done in Drosophila. Nonetheless, if the change in polysome profile is pro-survival/anti-apoptotic and requires an optimal level of EF1-a, changes in the latter may have an effect on cell death. Second, mammalian EF1-a is known to have several unexpected binding partners including those with known survival/apoptotic roles such as Akt and TRADD [37], [38]. The role of Drosophila EF1-a in suppressing apoptosis may result from such an interaction. Thus, Finally, as mentioned in a preceding section, uneven protein synthesis capacity in neighboring cells is known to result in cell competition in which cells with lower capacity are eliminated through apoptosis (reviewed in [24]). Importantly, this mechanism has been proposed to underlie p53-independent apoptosis in irradiated Drosophila wing imaginal discs [9]. Here, chromosome breakage by irradiation is proposed to result in deletion of ribosomal protein/RNA loci in some cells, which then result in uneven protein synthesis capacity in neighbors and death through competition. Elevations in EF1–a levels we see in irradiated p53 mutants may help counteract cell competition and thus prevent apoptosis. More work will be needed to understand the role of EF1–a in apoptosis in Drosophila. We note, however, that a ribosomal protein, S27L, was shown to be induced by p53 in multiple human cancer cell lines, and is needed for apoptosis induced by a chemotherapy drug, etoposide; the mechanism remains unknown in this case also [39]. Related to this discussion, we find that a chemical inhibitor of translation elongation, a process that EF1-a acts in, can enhance the effect of radiation in human cancer cells and xenografts [40]. The mechanism for radiation enhancement needs to be determined, but it is clear that regulation of translation elongation plays an important role in radiation responses.

In summary, we propose that the role of p53 in inducing apoptosis after IR exposure is not only through transcriptional activation of pro-apoptotic genes such as *rpr*, but also through repression, directly or indirectly, of anti-apoptotic genes such as E2F2 and EF1–a 100E. In the absence of p53, anti-apoptotic activities are not repressed and act to inhibit apoptosis at shorter times after irradiation. At longer times after irradiation, pro-apoptotic activities such as those contributing to the JNK cascade accumulate sufficiently to counterbalance anti-apoptotic activities, leading to cell death. Imbalances in ribosome function may contribute to promote cell death by activating apoptotic genes such as *hid* through cell competition [24]. If would be interesting to see if a similar situation exists in mammalian cells, with multiple inputs, both positive and negative, collaborating to induce p53-independent apoptosis in response to IR. The presence of multiple inputs could mean that there are multiple drug targets to choose from in efforts to improve radiotherapy of p53-deficient tumors.

## Materials and Methods

### Drosophila stocks

Flies were raised under standard conditions at 25°C. Wild type flies were of the *y^1^w^1118^* stock. *p53^5A-1-4^* is a targeted deletion allele and is used as *y^1^w^1118^*; *p53^5A-1-4^*. *y^1^w^67c23^*; *Ef1a100E^EY20714^* was used as Ef1-a100E mutant; this allele results from a p-element insertion at the junction of intron 1 and exon 2 (http://flybase.org/). Chromosomal deficiency was Df(3R)BSC505. p53 RNAi line (#38235) was obtained from Vienna Drosophila RNAi Center and was recombined with *engrailed*-GAL4>UAS-GFP transgenes on Chromosome II using standard techniques.

### Microarrays

#### Tissue Collection

Embryos were collected for 4 hours and aged at 25°C for 118 hours to reach 120±2 hr in age. Feeding third instar larvae were exposed to 4000R of X-rays in a TORREX X-ray generator (Astrophysics Research), set at 115 kV and 5 mA. 60 wing discs per sample were dissected in PBS, 2–3 or 18–19 hours post irradiation, and stored at −80°C. Non-irradiated wing disc were dissected from age-matched larvae for control.

#### RNA isolation

Total RNA from wing discs was isolated using the RNeasy Plus kit (Qiagen). RNA integrity of one representative sample was determined by analyzing the 18S and 28S ribosomal protein bands on a 1% agarose gel. Purity of all RNA samples was determined by the 260/280 ratios using a NanoDrop 1000 Spectrophotometer (Thermo Scientific). Isolated RNA was stored at −80°C.

RNA labeling and microarray processing: The Genechip® 3′ IVT Express Kit (Affymetrix) was used to reverse transcribe the RNA and to in vitro transcribe the resulting cDNA into Biotinyl labeled RNA (aRNA). aRNA was purified and the quality and concentrations were assessed as described in the preceding paragraph. aRNA was fragmented and hybridized to a GeneChip Drosophila Genome 2.0 Array (Affymetrix). GeneChip's were washed, stained and scanned. All steps were performed to according to manufacturer's instructions.

Microarray analysis was performed with the R statistical environment version 2.12.2 using the Bioconductor package [41]. The GCRMA method with default options was used for normalization, background correction and summarization across all microarrays [42], [43]. P-values for each probe set were computed across microarray groups using the Cyber-T function bayesT [44]. The Cyber-T statistical method for assessing differential expression was used because it has been shown to partially compensate for a lack of replication [44], and has been shown to outperform other common methods using spiked-in datasets [45], [46]. Gene Ontology analysis was performed using the DAVID (Database for Annotation, Visualization and Integrated Discovery) functional annotation online analysis tool [20].

### Quantitative-RT-PCR

To confirm microarray results, an aliquot of the same RNA sample used for the microarray analysis was used for the Q-RT-PCR. 1 µg RNA was reverse transcribed into cDNA by using the iScript cDNA synthesis kit (Bio-Rad). Primers for the Q-RT-PCR were designed against a sequence in the same exon as the sequence covered by the probe set on the Affymetrix gene chip by using the Integrated DNA Technologies (DNT) SciTools PrimerQuestSM. PCRs containing SYBR Green Mix (Applied Biosystems), 5.0 ng of cDNA (candidate genes) or 0.3 ng cDNA (α-Tubulin), and 500 nM primers were set up and read in a 7900HT RT-PCR instrument (Applied Biosystems). Relative levels of cDNA of our candidate genes among different conditions were determined by using a standard curve for each set of primers. α-tubulin levels were used to for normalization and non-reversed transcribed RNA was used to correct for the presence of genomic DNA. Detailed protocols for Q-RT-PCR are available upon request.

Primer sequences (Fw = forward, Rv = reverse):

a-tubulin 84B Fw,TCCAATCGCAACAAAAAATTCA

a-tubulin 84B Rv,TCGTTTTCGTATGCTTTTCAGTGT

Tsp42edFw,ATTACGGCATTACGTATCCCGCCT,

Tsp42edRv,ACGTTGGTGTCCCAGAAGGAATCA,

Cyp6d2Fw,ATTCCTCAATCGAGAGTGCACCGA,

Cyp6d2Rv,TGCATGCCAAAGAGCGAGATCAGA,

EigerFw,ACTCGCACGACCAGAACGGATTTA,

EigerRv,GGTGTGCACCTTATGTGGCATGTT,

Impl3Fw,TGACAAGGATGTGTTCCTCTCGCT,

Impl3Rv,ATCGGACATGATGTTGGCGGACTT,

Ef1a100eFw,TTCCGAGATCAAGGAGAAGTGCGA,

Ef1a100eRv,TCCTGGAAGCTCTCTACGCACAA,

CG2064Fw,AGACCTCGATTTACGCTGCTTTGG,

CG2064Rv,ATCTAATCCGGTCCACTTCTCGCT,

GADD45Fw,GCCATCAACGTGCTCTCCAAGT,

GADD45Rv,CACGTAGATGTCGTTCTCGTAGCA,

Spn-eFW,TGGGAAACCAATCCCGAACTACCA,

Spn-eRv,TGCAGTTCTCTCTCAGTTGCACCA,

Traf4(2)Fw, ACACAGGCACTCTGTTGTGGAAGA,

Traf4(2)Rv, ATGTAGACGGAGACGTGCGTGTTT,

CG9411(3)Fw,TATGGTCCACCGCCATCTGGAAAT,

CG9411(3)Rv,ATTCAGCTGGATGCTCTGCGACTT,

### Caspase staining and image analysis

Larval imaginal discs were dissected in PBS, fixed in PBS+4% formaldehyde, and stained for active cleaved Caspase 3 as described in before [8]. Primary antibody was rabbit anti-cleaved Caspase 3 (Cell Signaling Cat#9661, lot 32) used at 1∶100. Secondary antibody was anti-rabbit Rhodamine-conjugated used at 1∶500 final dilution (Jackson).

Images were acquired on PerkinElmer Spinning Disc Confocal attached to a Leica DMR compound microscope, using MetaMorph software (Molecular Devices). For each disc, at least 20 Z-sections 1 µm apart were acquired and collapsed in ImageJ (NIH). Caspase signal was quantified by manually selecting the area using GFP signal as a guide and fluorescence intensity measured in ImageJ. Collapsed Z-stacks were also saved as JPEG files and assembled into figures in Powerpoint without further manipulation except as noted in figure legends. The significance for signal comparisons was calculated using a two-tailed t-test.

## Supporting Information

Figure S1 **A Relative Log Expression (RLE) plot shows that all arrays used were of similar quality.** Samples were, from left to right: “p53^−/−^,18 hr, −IR” “p53^−/−^,18 hr, +IR” “p53^−/−^,2 hr, −IR” “p53^−/−^,2 hr, +IR” “wt,2 hr −IR” “wt,2 hr +IR” “wt,18 hr −IR” “wt,18 hr +IR”. Lower quality arrays are indicated by more spread out boxes.
(TIF)

Figure S2 **A Normalized Unscaled Standard Errors (NUSE) plot (B) shows that all arrays used were of similar quality.** Samples were, from left to right: “p53^−/−^,18 hr, −IR” “p53^−/−^,18 hr, +IR” “p53^−/−^,2 hr, −IR” “p53^−/−^,2 hr, +IR” “wt,2 hr −IR” “wt,2 hr +IR” “wt,18 hr −IR” “wt,18 hr +IR”. Lower quality arrays are indicated by more spread out boxes.
(TIF)

Figure S3 **The plot of expression values of each gene in duplicate samples shows that most expression values are similar in both arrays.** Expression values for the first array experiment were plotted against the expression values for the second array experiment for any given sample.
(TIF)

Figure S4 **Venn diagrams to show overlap in gene expression changes.** The data are from Table S1, which shows genes that with ±1.5-fold or greater change (p<0.005) between ANY ±IR sample pairs at 2 or 18 hr, in *yw* or *p53* discs. Gene ontology information is from DAVID (Database for Annotation, Visualization and Integrated Discovery) Bioinformatics Resources 6.7, NIAID/NIH (ttp://david.abcc.ncifcrf.gov/). Examples of genes in each category are shown.
(TIF)

Figure S5 **EF1a mutants show elevated levels of IR-induced apoptosis in a p53-depleted background.** Wing imaginal discs were dissected from 3^rd^ instar larvae at 24 hr after exposure to 0 (−IR) or 4000R (+IR) of X-rays. Apoptosis was detected by staining with an antibody to active cleaved Caspase 3. GFP boundary is used to mark the boundary between anterior and posterior compartments. *en-GAL4* is active only in the posterior compartment. (A and B) p53^RNAi^ = *en-GAL4>UAS-dsRNA* against p53, *UAS-GFP*. Caspase stain is in (A) and GFP fluorescence is in (B). (C and D) p53^RNAi^, EF1-a = same as in (A) but in trans-heterozygotes of *Ef1a100E^EY20714^* and a chromosomal deficiency that removes the EF1-a gene. Un-irradiated control discs stained for caspase, to show little or no apoptosis in the absence of irradiation.
(TIF)

Table S1 **Genes that show significant induction or repression (p<0.005, ±1.5-fold or greater) in any one of the following pair-wise comparisons: y2− vs y2+; y18− vs y18+; p2− vs p2+; p18− vs p18+; p2+ vs p18+.** y = *yw* control; p = *p53* mutants, ‘−’ = −IR (0 R); ‘+’ = +IR (4000 R); 2 = 2 hr after irradiation, 18 = 18 hr after irradiation. “p53-independent candidate” refers to candidate regulators of p53-independent apoptosis. These genes show significant induction in p18− vs p18+ comparison, significant induction in p2+ vs p18+ comparison. These genes may show significant induction in p2− vs p2+ comparison, but fold-induced in p18− vs p18+ comparison has to be greater than fold induced in p2− vs p2+ comparison for a gene to be included in this category. Genes that fall within the intersect of selected sample pairs as indicated in column headings are marked as such. “Intersect p18 & y18” means genes that are significantly changed (induced or repressed) at 18 hr after IR in both *p53* and *yw* compared to age-matched −IR controls; “Intersect p18 & y2 & y18” means genes that show significant change (induced or repressed) by IR at 18 hr in *p53*, at 2 hr in *yw* and 18 hr in *yw*; and so on. Gene ontology information is from DAVID Bioinformatics Resources 6.7, NIAID/NIH (ttp://david.abcc.ncifcrf.gov/). p-value of 0 means p<1e-10.
(XLS)

Table S2 **Genes that show significant induction (p<0.001, 2-fold or more) in** ***yw*** **discs at 2 and 18 hr after irradiation.** If there is data for more than one probe set is available for a gene, the set with the best p value was considered. Genes that show induction at both 2 and 18 hr time points are in bold. p-value of 0 means p<1e-10.
(XLS)

Table S3 **Cell death-related genes repressed by IR in wild type imaginal discs.** The values shown are log2. The cut-off values were 1.5 fold or more (log2 of 0.585 or greater) with p<0.005 compared to un-irradiated controls, at 2 hr or 18 hr after irradiation or both. ‘-’ = the gene was not significantly induced with respect to neither p-value or fold change. p-value of 0 means p<1e-10. y = yw control; p = p53 mutants, ‘−’ = −IR (0 R); ‘+’ = +IR (4000 R); 2 = 2 hr after irradiation, 18 = 18 hr after irradiation. If there is data for more than one probe set is available for a gene, the set with the best p value was considered.
(XLS)

Table S4 **Genes induced by IR in wing imaginal discs of** ***p53*** **mutants.** 87 genes whose expression in *p53* mutants (1) increased significantly at 18 hr after irradiation compared to un-irradiated *p53* mutants (p<0.005 and fold change of 1.5 or greater), and (2) showed a significant increase in IR+18 hr *p53* mutant compared to IR+2 hr *p53* mutant (p<0.005 and fold change of 1.5 or greater; schematic in Figure 1A).
(XLS)
