# Supplementary figures and images for: Genome-Wide Expression Analysis Identifies a Modulator of Ionizing Radiation-Induced p53-Independent Apoptosis in Drosophila melanogaster
Source: PLoS One. 2012 May 29;7(5):e36539. doi: 10.1371/journal.pone.0036539 (PMC3362589; doi:10.1371/journal.pone.0036539)

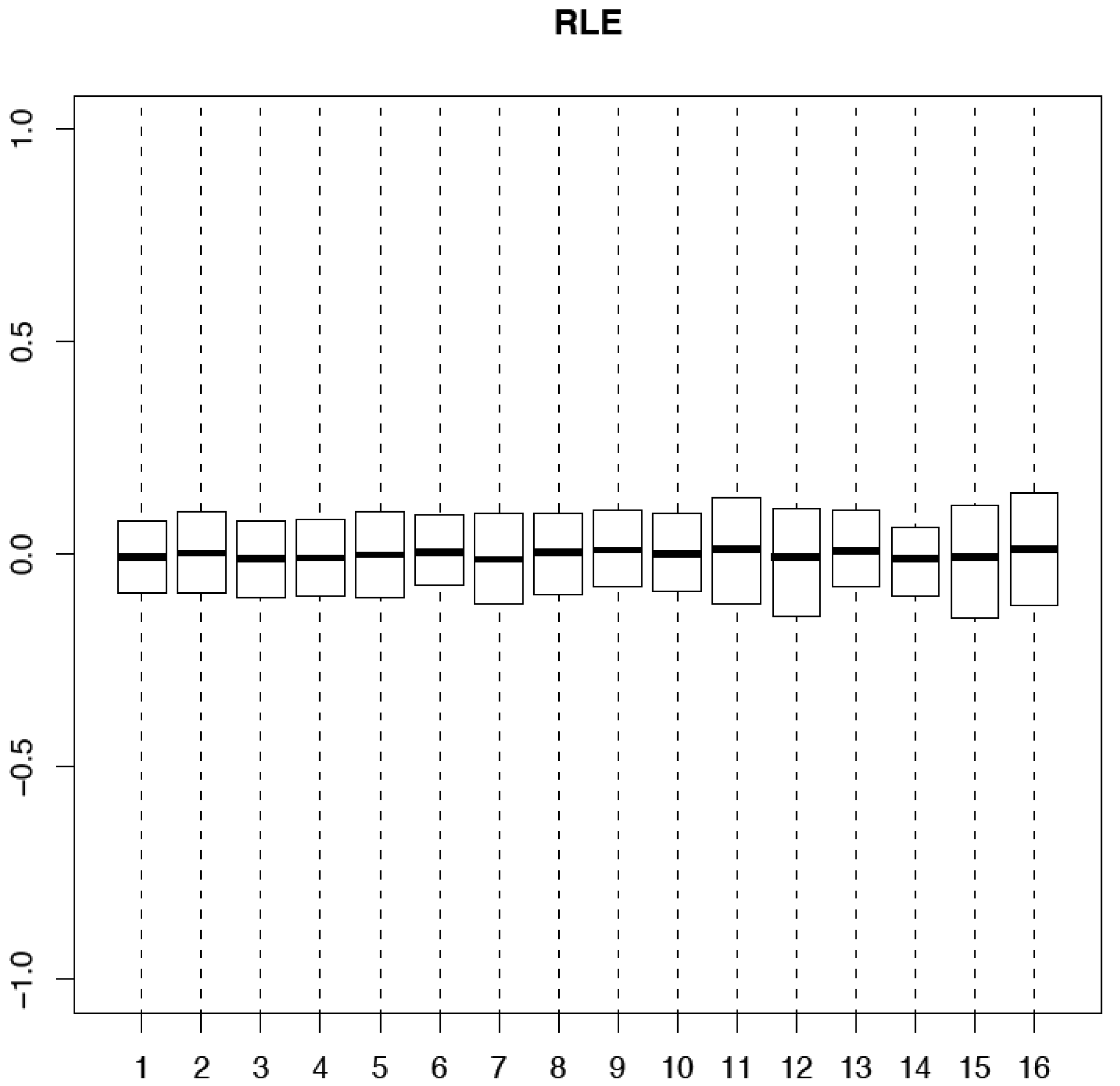

Supplement: Figure S1 — A Relative Log Expression (RLE) plot shows that all arrays used were of similar quality. Samples were, from left to right: “p53−/−,18 hr, −IR” “p53−/−,18 hr, +IR” “p53−/−,2 hr, −IR” “p53−/−,2 hr, +IR” “wt,2 hr −IR” “wt,2 hr +IR” “wt,18 hr −IR” “wt,18 hr +IR”. Lower quality arrays are indicated by more spread out boxes. (TIF) [file pone.0036539.s001.tif]

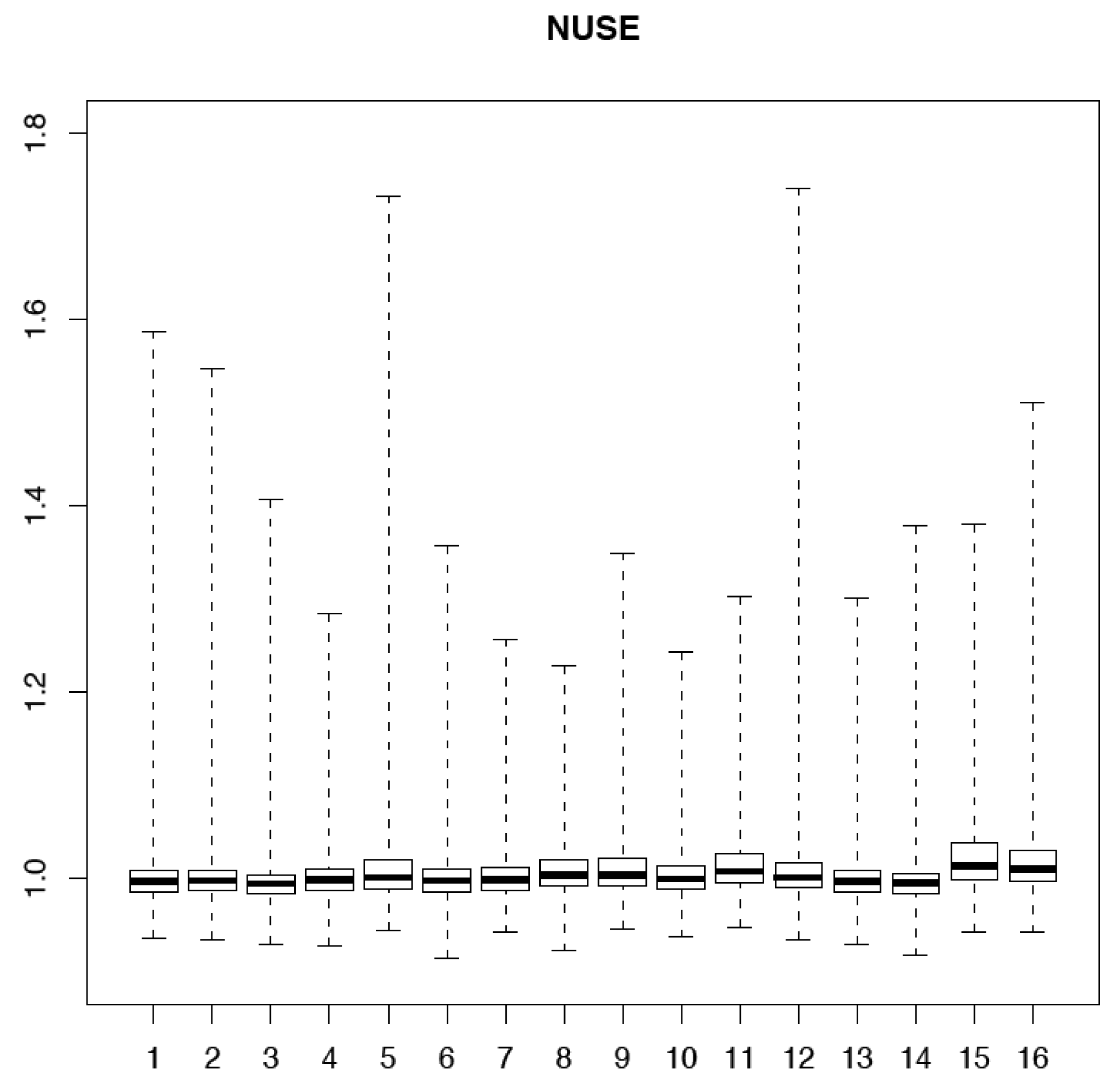

Supplement: Figure S2 — A Normalized Unscaled Standard Errors (NUSE) plot (B) shows that all arrays used were of similar quality. Samples were, from left to right: “p53−/−,18 hr, −IR” “p53−/−,18 hr, +IR” “p53−/−,2 hr, −IR” “p53−/−,2 hr, +IR” “wt,2 hr −IR” “wt,2 hr +IR” “wt,18 hr −IR” “wt,18 hr +IR”. Lower quality arrays are indicated by more spread out boxes. (TIF) [file pone.0036539.s002.tif]

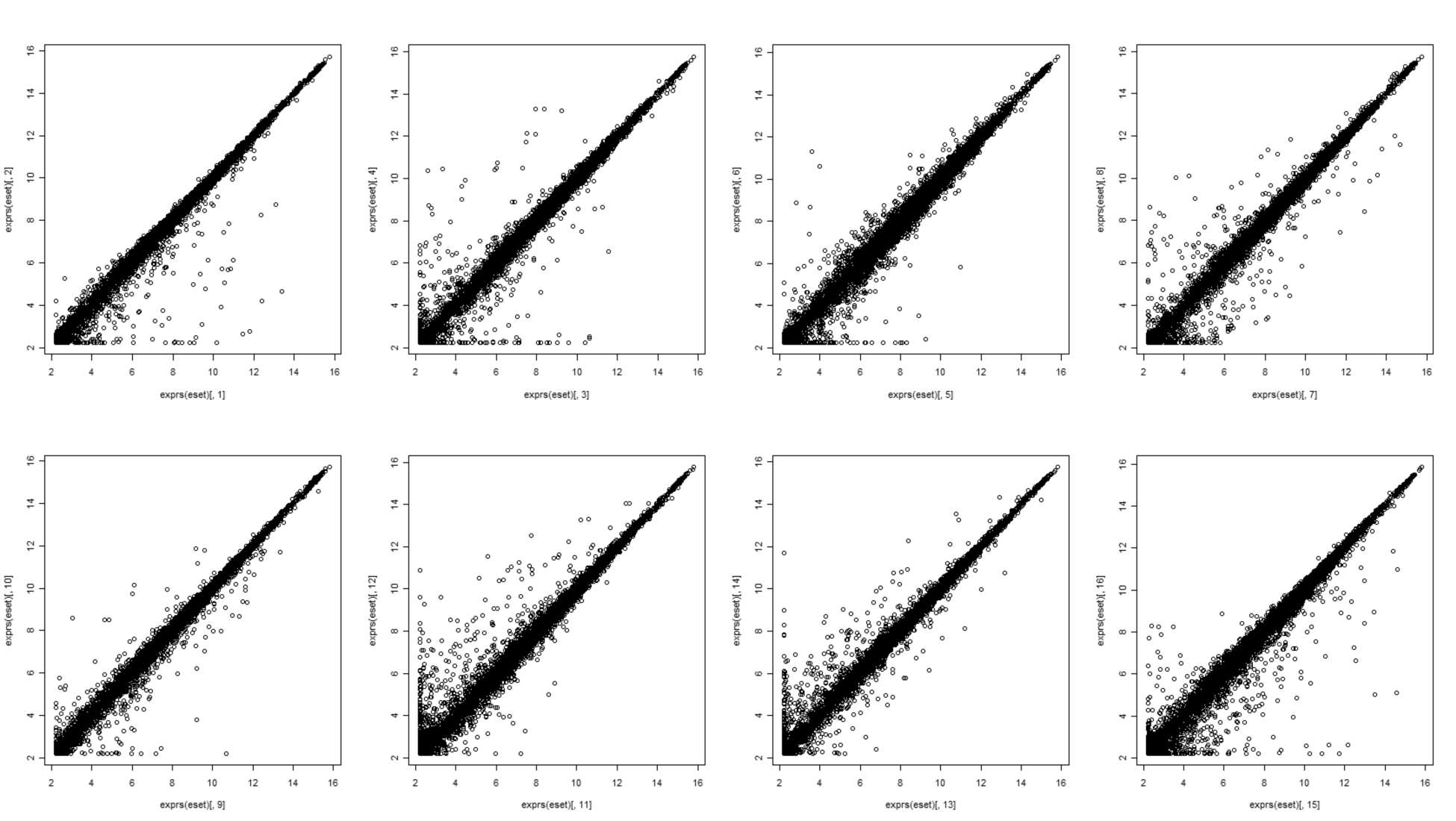

Supplement: Figure S3 — The plot of expression values of each gene in duplicate samples shows that most expression values are similar in both arrays. Expression values for the first array experiment were plotted against the expression values for the second array experiment for any given sample. (TIF) [file pone.0036539.s003.tif]

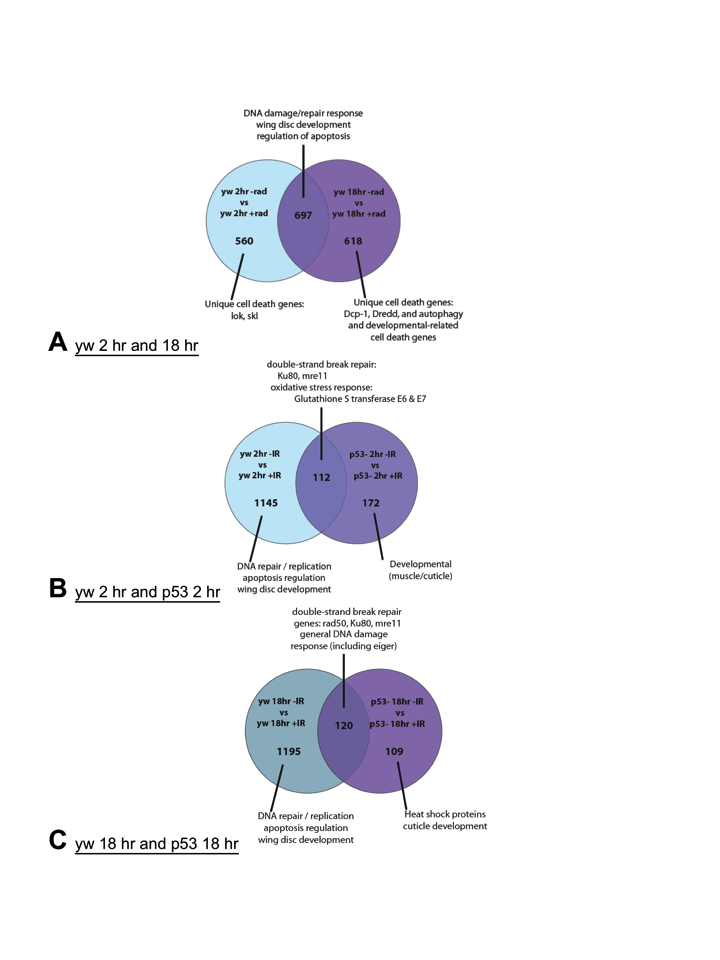

Supplement: Figure S4 — Venn diagrams to show overlap in gene expression changes. The data are from Table S1, which shows genes that with ±1.5-fold or greater change (p<0.005) between ANY ±IR sample pairs at 2 or 18 hr, in yw or p53 discs. Gene ontology information is from DAVID (Database for Annotation, Visualization and Integrated Discovery) Bioinformatics Resources 6.7, NIAID/NIH (ttp://david.abcc.ncifcrf.gov/). Examples of genes in each category are shown. (TIF) [file pone.0036539.s004.tif]

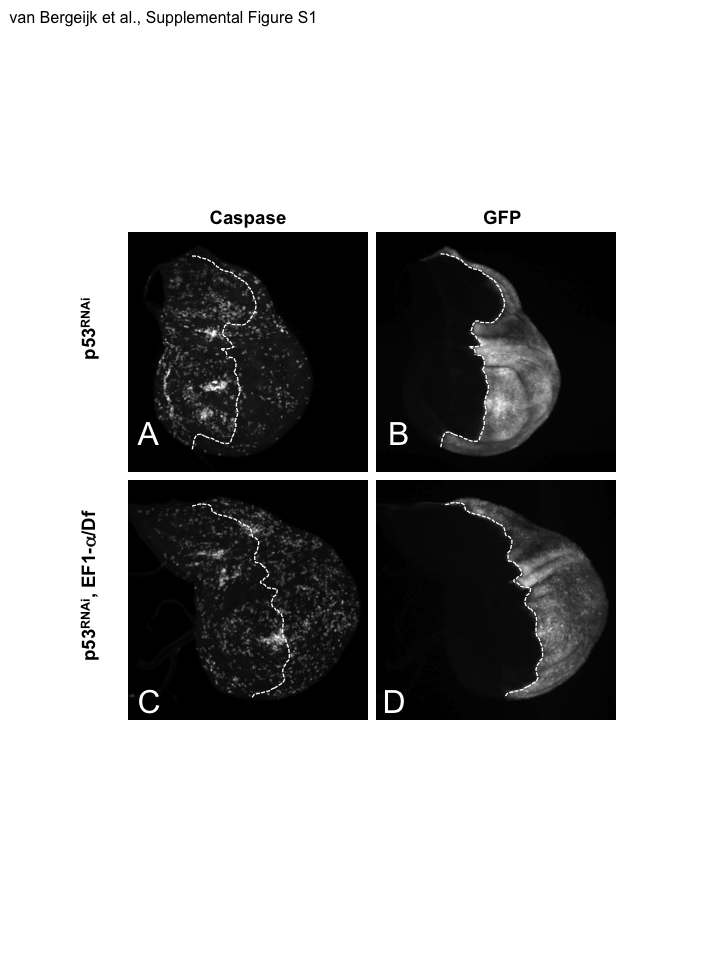

Supplement: Figure S5 — EF1a mutants show elevated levels of IR-induced apoptosis in a p53-depleted background. Wing imaginal discs were dissected from 3rd instar larvae at 24 hr after exposure to 0 (−IR) or 4000R (+IR) of X-rays. Apoptosis was detected by staining with an antibody to active cleaved Caspase 3. GFP boundary is used to mark the boundary between anterior and posterior compartments. en-GAL4 is active only in the posterior compartment. (A and B) p53RNAi = en-GAL4>UAS-dsRNA against p53, UAS-GFP. Caspase stain is in (A) and GFP fluorescence is in (B). (C and D) p53RNAi, EF1-a = same as in (A) but in trans-heterozygotes of Ef1a100EEY20714 and a chromosomal deficiency that removes the EF1-a gene. Un-irradiated control discs stained for caspase, to show little or no apoptosis in the absence of irradiation. (TIF) [file pone.0036539.s005.tif]
